# Topics and Sentiments of Public Concerns Regarding COVID-19 Vaccines: Social Media Trend Analysis

**DOI:** 10.2196/30765

**Published:** 2021-10-21

**Authors:** Michal Monselise, Chia-Hsuan Chang, Gustavo Ferreira, Rita Yang, Christopher C Yang

**Affiliations:** 1 College of Computing and Informatics Drexel University Philadelphia, PA United States; 2 Department of Information Management National Sun Yat-sen University Kaohsiung Taiwan; 3 Virtua Voorhees Hospital Voorhees Township, NJ United States

**Keywords:** health care informatics, topic detection, unsupervised sentiment analysis, COVID-19, vaccine hesitancy, sentiment, concern, vaccine, social media, trend, trust, health information, Twitter, discussion, communication, hesitancy, emotion, fear

## Abstract

**Background:**

As a number of vaccines for COVID-19 are given emergency use authorization by local health agencies and are being administered in multiple countries, it is crucial to gain public trust in these vaccines to ensure herd immunity through vaccination. One way to gauge public sentiment regarding vaccines for the goal of increasing vaccination rates is by analyzing social media such as Twitter.

**Objective:**

The goal of this research was to understand public sentiment toward COVID-19 vaccines by analyzing discussions about the vaccines on social media for a period of 60 days when the vaccines were started in the United States. Using the combination of topic detection and sentiment analysis, we identified different types of concerns regarding vaccines that were expressed by different groups of the public on social media.

**Methods:**

To better understand public sentiment, we collected tweets for exactly 60 days starting from December 16, 2020 that contained hashtags or keywords related to COVID-19 vaccines. We detected and analyzed different topics of discussion of these tweets as well as their emotional content. Vaccine topics were identified by nonnegative matrix factorization, and emotional content was identified using the Valence Aware Dictionary and sEntiment Reasoner sentiment analysis library as well as by using sentence bidirectional encoder representations from transformer embeddings and comparing the embedding to different emotions using cosine similarity.

**Results:**

After removing all duplicates and retweets, 7,948,886 tweets were collected during the 60-day time period. Topic modeling resulted in 50 topics; of those, we selected 12 topics with the highest volume of tweets for analysis. Administration and access to vaccines were some of the major concerns of the public. Additionally, we classified the tweets in each topic into 1 of the 5 emotions and found fear to be the leading emotion in the tweets, followed by joy.

**Conclusions:**

This research focused not only on negative emotions that may have led to vaccine hesitancy but also on positive emotions toward the vaccine. By identifying both positive and negative emotions, we were able to identify the public's response to the vaccines overall and to news events related to the vaccines. These results are useful for developing plans for disseminating authoritative health information and for better communication to build understanding and trust.

## Introduction

### Background

In late 2020, the COVID-19 pandemic had approached the year mark when a number of pharmaceutical companies began to release their vaccine clinical trial results. A global sense of relief was felt when the results of the clinical trials looked promising. The first vaccine developed by Pfizer and BioNTech was given for emergency use authorization in December 2020 by the US Food and Drug Administration [1]. While this timeline seemed too fast for some, most vaccines for COVID-19 relied on many years of previous scientific work. For example, mRNA-based vaccines had been in development for over a decade at that point [2-4]. Despite efforts of the scientific community to assure the public that these vaccines are safe and effective, public sentiment has been mixed. There has been a significant amount of public hesitancy toward vaccination against COVID-19 [5]. At the same time, many have expressed excitement over the prospect of returning to a prepandemic world. Given this mixed reaction, it is essential to investigate the actual public sentiment regarding COVID-19 vaccines. Particularly, we were interested in learning about public sentiment for a period of 60 days when the vaccines were started in the United States. Social media provides a great data source for listening to the public on what they are thinking and what concerns and questions they have. We used Twitter as a proxy for public sentiment and were able to find the most important discussion topics that pertained to COVID-19 vaccines in the early days of the vaccine rollout. Additionally, we were able to classify public sentiment as it pertained to the vaccines and how this sentiment changed over time overall and in each topic as well. The goal of this research was to examine the discussion topics and public sentiment toward COVID-19 vaccines. By studying the topic and sentiment of the discussion on COVID-19 vaccines on Twitter, we may understand public concerns as they happen and learn more accurately about the source of vaccine hesitancy. By learning what drives vaccine hesitancy, we can better address it and formulate tailored and targeted communication. Conversely, we may also learn about the excitement toward the vaccine and study what is going well and what resonates well with the public on social media. This research will use the results uncovered by the topic and sentiment analysis of the Twitter data and suggest actionable insights for practitioners to address COVID-19 vaccine hesitancy. This research will also address how to utilize positive sentiment toward the vaccine.

### Previous Works

#### Public Sentiment on COVID-19 Vaccine

A number of studies about vaccine hesitancy on social media have been published during the pandemic. Before any vaccine was approved, research showed hesitancy on social media. Harrison and Wu [6] examined vaccine hesitancy at the start of the pandemic and discussed methods to reduce vaccine hesitancy in preparation for the vaccine that would eventually come. This paper critiques current approaches for combating vaccine hesitancy with the goal of improving on these approaches when the COVID-19 vaccines are authorized for emergency use. A study by Chou and Budenz [7] discusses both methods for reducing hesitancy as well as for fostering positive emotions toward the vaccine. They propose acknowledging fear, anger, and other negative emotions and addressing them to convince the public to get vaccinated. A study by Wilson and Wiysonge [8] showed the existence of organized disinformation campaigns against the vaccines for COVID-19. However, this study focused on exposing negative sentiment against the vaccine and did not measure the positive sentiment toward the vaccine on social media. While the abovementioned studies discuss public sentiment, they do not measure both positive and negative sentiment, and some just make recommendations rather than looking at empirical evidence.

#### Topic Detection in COVID-19–Related Tweet Sentiment Analysis

Owing to the pandemic and quarantine policy, social media platforms such as Twitter became the main channel for people to share thoughts and to express their opinions about any impacts caused by the COVID-19. The hidden topics underneath such massive textual contents on social media help governments and health care units to understand the demand of the general public so as to make better decision and quick response. Cinelli et al [9] extracted topics using Partitioning Around Medoids algorithm on word vector representations and proposed a custom epidemic model for characterizing misinformation spreading speed in different social platforms. Since the temporal trends of the hidden topics reflect concerns of the general public through time, Chang et al [10] proposed 2 temporal models based on nonnegative matrix factorization (NMF), which help to identify the trends of several important themes such as government policy, economic crisis, COVID-19 case updates, COVID-19 urgent events, prevention, vaccines and treatments, and COVID-19 testing.

#### Sentiment Analysis

Sentiment analysis is a research area that involves the classification of text, images, or audio into a set of one or more sentiments [11]. In the context of this research, we will be classifying the sentiment of short snippets of text. When classifying text, we can classify at the word, sentence, or document level. There are different classification methods, including rule-based [12-14], support vector machine [15,16], random forest [17], Naive Bayes [18,19], embedding-based [20,21], as well as sentiment analysis using neural networks [22-25]. Additionally, we may classify sentiment by using unsupervised methods such as methods using rule-based unsupervised sentiment analysis [26], embeddings such as Word2Vec and Doc2Vec [27], and lexical resources for sentiment analysis [28].

### Sentiment Analysis in Twitter

Sentiment analysis is an established research field in the area of natural language processing. However, performing sentiment analysis on tweets is a slightly different task. Zimbra et al [29] reviewed a number of techniques for classifying sentiment in tweets. They found that due to factors such as the brevity of tweets, Twitter-specific language [30], and a class imbalance [31], classification algorithms achieved an accuracy of around 70%. However, Adwan et al [32] also reviewed a large number of techniques and they found a mix of accuracy scores, with some papers passing 80% accuracy while others still perform below 80% even with new algorithms [33]. Among those who have improved their accuracy, some only focus on specific politics-related data sets [34], some propose methods that require a large number of steps [35], while others address the issues with tweets, such as Twitter-specific language [36].

## Methods

**Figure 1:**
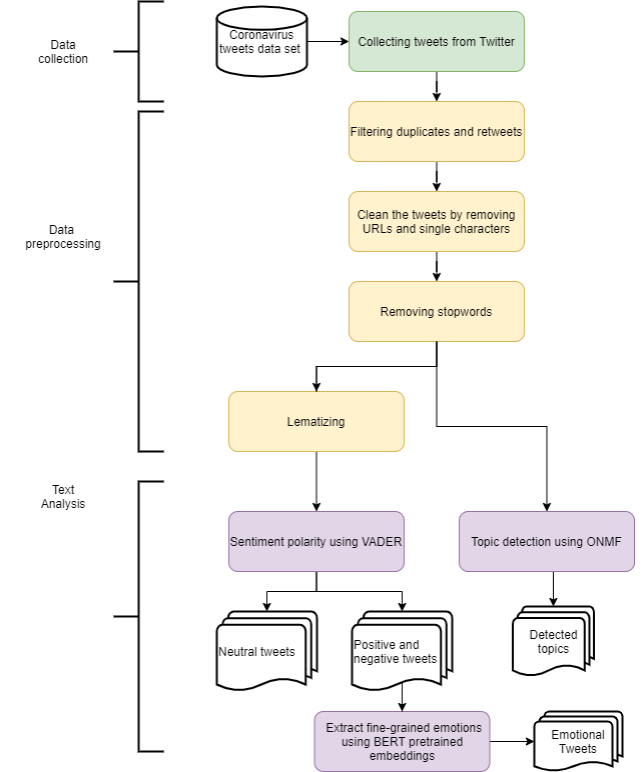
Pipeline of our text analysis. BERT: bidirectional encoder representations from transformers; ONMF: online nonnegative matrix factorization; VADER: Valence Aware Dictionary and sEntiment Reasoner.

Our entire pipeline is described in Figure 1. We first introduce the data collection and preprocessing. We then detail our topic detection algorithm and procedure of sentiment and emotion classification.

### Data Collection

We adopted the coronavirus tweets data set [37] as our data source, which uses over 90 keywords and hashtags [38] to monitor the real-time coronavirus-related tweets from February 05, 2020 till present. Since the US Food and Drug Administration authorized Pfizer-BioNTech COVID‑19 vaccine and Moderna vaccine for emergency use in mid-December, we only kept tweets that were created during a 60-day period between December 16, 2020 and February 13, 2021 for extracting discussion topics and their sentiment from the general public about COVID-19 vaccines. Owing to the data sharing policy of Twitter, the coronavirus tweets data set only shares the IDs of the collected tweets. Therefore, we employed Twitter’s tweet lookup application programming interface [39] to retrieve the content and metainformation of each retained tweet. In order to downsize the corpus and retain vaccine-related tweets, we only selected tweets that contained at least 1 keyword in our predefined keyword list: “vaccine,” “vaccines,” “#vaccine,” “#vaccines,” “corona vaccine,” “corona vaccines,” “#coronavaccine,” “#coronavaccines,” “pfizer,” “biontech,” “moderna,” “Pfizer-BioNTech,” “Pfizer/BioNTech,” “Pfizer BioNTech,” “#PfizerBioNTech,” “COVAX,” “COVAX,” “Sinopharm,” “Sinovac,” “AstraZeneca,” “Sputnik V,” and “Gamaleya.” The list of keywords was generated by the authors with the intention of collecting data on COVID-19 vaccines in general as well as the specific vaccines that were available to the public at the start of the data collection period. We also filtered out duplicated content, for example, retweets and non-English contents for providing more consistent data. Thus, we had 7,948,886 tweets for further text analysis.

### Topic Detection

There are 2 types of models for topic detection: latent Dirichlet allocation [40] and NMF [41]. In this study, we chose NMF because its superiority has been proved in extracting topics from tweets [42]. NMF is a matrix factorization algorithm that learns and maps high-dimensional data into low-dimension representations. In this study, our tweet corpus V ∈ 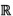
^F×N^ is represented as a matrix with rows (words) and columns (tweets). After the preprocessing process detailed in Figure 1, we constructed the corpus using tf-idf weighting scheme:



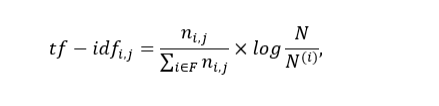



Where *n_i,j_* is the count of word *i*∈*F* appearing in tweet *j*∈*N*, and *N^(i)^* is the number of tweets containing word *i*. With such weighting scheme, the word has more weights, as it is an important word for a tweet. After encoding the corpus, we apply NMF for extracting topics, whose objective of factorization is as follows:



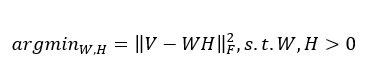



We can exploit the topic word distribution using *W*∈ *
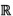
^K×N^* because each column represents a hidden topic, where the representative words will be encoded more weights. *H*∈ *
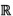
^K×N^* can be served as document topic distribution since each column indicated a topic weight distribution of each tweet. For coping with the large-scale tweets and the subsequent memory issue, we adopted online NMF (ONMF) [10,43] to solve both and in an online learning fashion. Specially, the whole tweet corpus will be divided into a set of small batches (
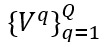
) and be sequentially used for updating *W^q^* and *H^q^* of each batch. The step for updating the coefficient of the current batch *H^q^* is to fix the word dictionary of previous batch *W^q–1^* and find a *H* that recovers *W^q^* with least error (see line 6 in Algorithm 1). Similarly, to update the dictionary of the current batch *W^q^*,*H^q^* is then fixed, and the best *W* is solved using line 8 in Algorithm 1. The mathematical details of the 2 updating forms can be seen in Zhao and Tan [43]. As a result, Algorithm 1 is the whole procedure for topic detection.



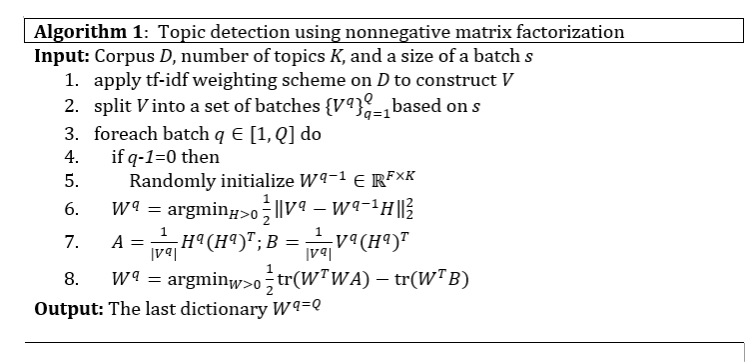



Note that we will use the final topic word dictionary *W^q=Q^* to infer topic weights of each tweet (ie, *H*). The representative topic of a tweet is determined by selecting the topic with maximum weight: argmax*_k_*_∈_*_K_H_k,j_* and we recorded the representative topics of all tweets as *H^rep^*∈ *
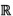
^1×N^*.

### Sentiment Analysis

To detect the sentiment conveyed in the tweets, we utilized a two-step approach. In the first step, we computed the polarity score of our tweets, and based on this score, we classified the tweets as either positive, neutral, or negative. In the second step, we classified the emotional content of the tweet into 1 of the 5 emotions: anger, fear, joy, hopefulness, and sadness.

#### Polarity Classification

The first classification step was performed using the VADER (Valence Aware Dictionary and sEntiment Reasoner) Python library [14]. The VADER library is a rule-based model for general sentiment analysis. VADER is constructed using existing well-established sentiment lexicons such as Linguistic Inquiry and Word Count and supplemented using lexical features commonly used to express sentiment in social media. After expanding using social media lexical terms, VADER was then human validated and is currently considered a gold standard in social media lexicons [44]. VADER evaluates the sentiment of each tweet by returning a compound sentiment score between –1 and 1. Based on the classification thresholds determined by the developers of the library, we assigned a negative sentiment to a compound score less than or equal to –0.05, a positive sentiment to all compound scores greater than or equal to 0.05, and a neutral sentiment to a compound score between –0.05 and 0.05 [14]. Since VADER is more sensitive to expressions of sentiment in the social media context, it performs better than other rule-based classification algorithms in this context [45]. It has been found that VADER outperforms individual human raters [14] in the F1 score.

#### Emotion Classification

In the second step, we separated our data into positive, negative, and neutral and detected 1 of the 2 emotions for positive polarity, that is, joy and hopefulness, and 1 of the 3 emotions for negative polarity, that is, anger, fear, and sadness. Since VADER only includes positive, negative, and neutral sentiment, to detect more fine-grained emotions, we used zero-shot classification, an unsupervised method for discovering the applicable emotion for each tweet. Zero-shot classification is used in machine learning to classify things such as images and text [46,47]. We detected the emotion by finding the BERT (bidirectional encoder representations from transformers) [48] embeddings of the tweets and of the emotion words (fear, joy, hopefulness, anger, and sadness) and then computing the cosine similarity of the emotion words and each tweet and selecting the emotion with the highest cosine similarity as the emotion associated with the tweet.

BERT [48] is a word representation model that uses unannotated text to perform various natural language processing tasks such as classification and question answering. By considering the context of a word using the words both before and after the word, we were able to produce embeddings for words that are more context aware. Our research used the pretrained sentence BERT [49] model to generate the embedding vectors for our emotion classification task.

Given our tweet corpus *V*∈ *
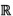
^F×N^*, we represented our emotion results as *X*∈ *
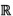
^C×N^*, where *C* is number of emotion categories and *N* is the number of tweets. For each emotion, we computed an embedding vector *Ec_i_* where *i =1,…..,C – 1* and for each tweet, we computed an embedding vector *Ev_j_* where *j = 1,……N* using the pretrained sentence BERT model. To populate our emotion matrix, we first computed the VADER sentiment score and assigned the score to the neutral category in our matrix. We then computed the cosine similarity between each of the remaining *C-1* categories and each tweet using the following equation:



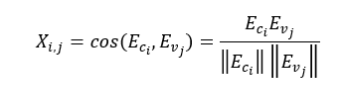



Where *i =1,…..,C – 1* and *j = 1,……N.* We assigned a representative emotion to each tweet by finding argmax_c∈_*_C_X_c,i_* for *i = 1,……N*, resulting into *X^rep^*∈ *
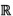
^1×N^*, which records representative emotions of all tweets.

### Combining Topic and Sentiment

We merged the detected topics *H^rep^*∈ *
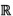
^1×N^* and identified emotions *X^rep^*∈ *
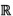
^1×N^* using the unique IDs of tweets, resulting into a matrix *O*∈ *
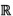
^2×N^*. By referring to the timestamp of each tweet, we were able to track the changes in the sentiment and topics over time to see how the public responded to the different vaccines as time passed.

## Results

### Tracking Topic Over Time

We started by generating 50 topics (*K*=50) using the ONMF algorithm with 2000 as the batch size (*s*=2000). In order to only retain the representative topics about vaccines, we calculated the ratio of each topic *k* using the following equation:



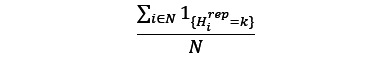



With the topic ratio, we could estimate how many tweets belonged to topic *k* and filtered out 38 insignificant topics whose topic ratios were below the average, that is, 2%. As listed in Table 1, the remaining 12 topics were then labeled by reviewing the most contributed keywords in each topic.

**Table 1:** The most significant 12 vaccine-related topics and the percentage of tweets in each topic (N=7,948,886).

Topic ID	Topic label	Topic totals, n (%)
1	Vaccination of frontline workers	690,357 (8.7)
2	Access to vaccines, signing up online	658,115 (8.3)
3	South African variant	593,425 (6.8)
4	Biden stimulus plan	540,065 (3.7)
5	mRNA vaccines	292,217 (3.2)
6	Complaints about pharmaceutical company profits	250,337 (3.1)
7	Vaccine conspiracy theories online	243,934 (2.9)
8	Trials in non-mRNA vaccines	232,780 (2.5)
9	Vaccine distribution in Canada	202,164 (2.5)
10	Supply and herd immunity	198,967 (2.5)
11	Genetic concerns about vaccines and kids	194,578 (2.2)
12	Low distribution of AstraZeneca vaccine	189,468 (2.1)

**Figure 2:**
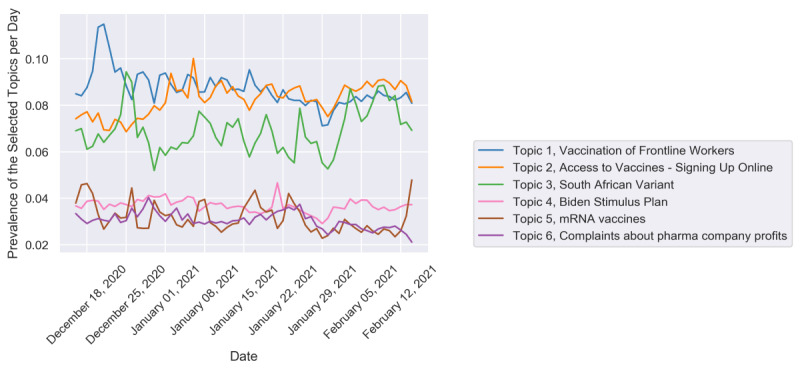
The topic trends for the most significant 6 topics that had a topic ratio above 3%.

**Figure 3:**
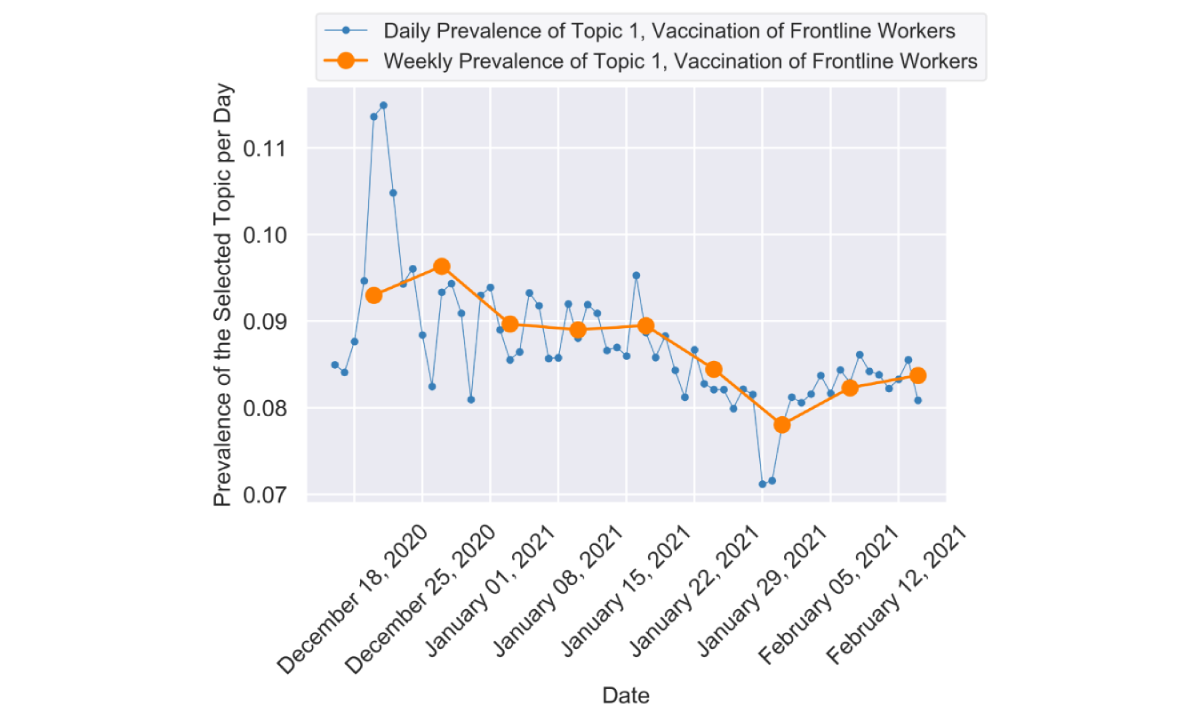
The daily and weekly trends of topic 1.

Figure 2 shows the trends for the 6 most important topics whose topic ratios were greater than 3%. The most important topic discussed the vaccination of frontline workers (topic 1), wherein the ratio stayed above 7% from mid-December to mid-February. Such a high attention of topic 1 indicated that people were concerned about the eligibility of vaccination and relevant plans from governments, especially in the early roll-out phases (ie, phase 1a and phase 1b). A discussion peak was observed on December 20, 2020, and December 21, 2020, as shown in Figure 3 because some congress members got vaccinated before frontline workers, which triggered heated debates. The representative tweets of topic 1 during that period were as follows:

…Speakers: Finding eligible #candidates for #COVID19 vaccine have to be ensured

December 20, 2020

…What makes Blumenthal and Murphy eligible for the vaccine. Are they frontline workers?

December 20, 2020

…They are depriving frontline workers of a vaccine. They are literally scum.

December 20, 2020

The above tweets reported that the priority of accepting COVID-19 vaccines and justice were also critical concerns of the people. The second largest topic was about access to vaccines—signing up online (topic 2). After the early distribution of the vaccines, we observed that people started to be concerned about the access to the vaccines, resulting in a growth starting from the last week of 2020. The following relevant tweets of topic 2 indicated that governments and health care facilities [50] began implementing online appointments for vaccination.

…Heads up Ottawa County-you can sign up for vaccine notifications online

May 1, 2021

…@drharshvardhan: Please implement Aadhaar-based online appointment for Covid vaccine as applicable in case of appointment for passport and driving license

May 1, 2021

…A step-by-step guide for the online vaccine appointment process

wenatcheeworld

The third largest topic was about the South African variant (topic 3), which peaked in late December and was relevant to the announcement of the South African variant from the South African health officials [51] and the first variant case detected in the United States [52], resulting in a rising trend from the late January of 2021. The high ratio of topic 3 indicated that the effectiveness of the released vaccines was of great concern, and people were skeptical and conservative. Finally, comparing the top 3 significant topics, topics 4-6 (ie, Biden Stimulus Plan, mRNA Vaccines, and Complaints about pharma company profits) showed relatively steady discussion trends.

**Figure 4:**
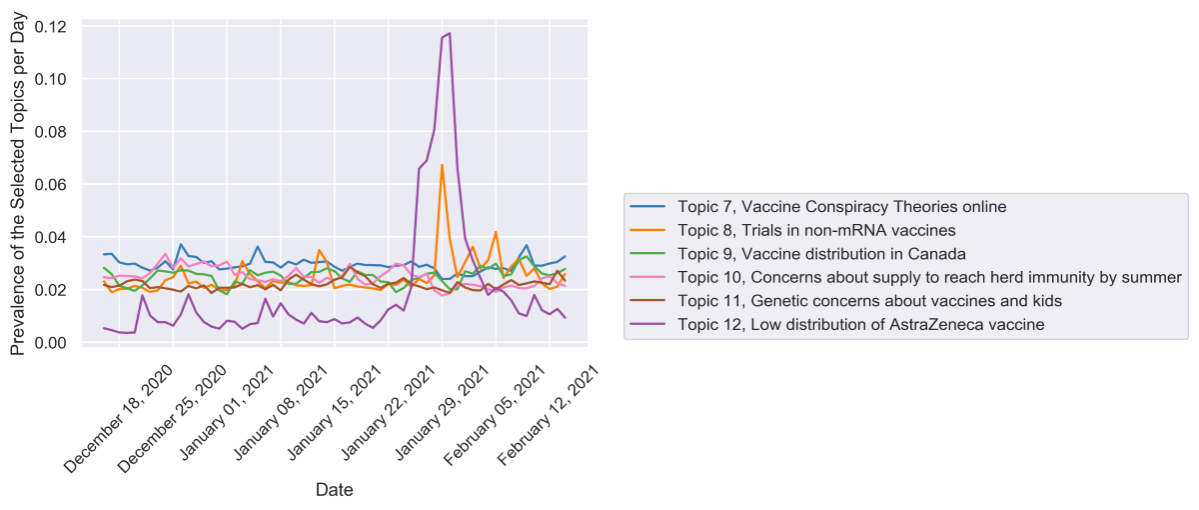
The topic trends for the rest of the topics that had a topic ratio below 3%.

**Figure 5:**
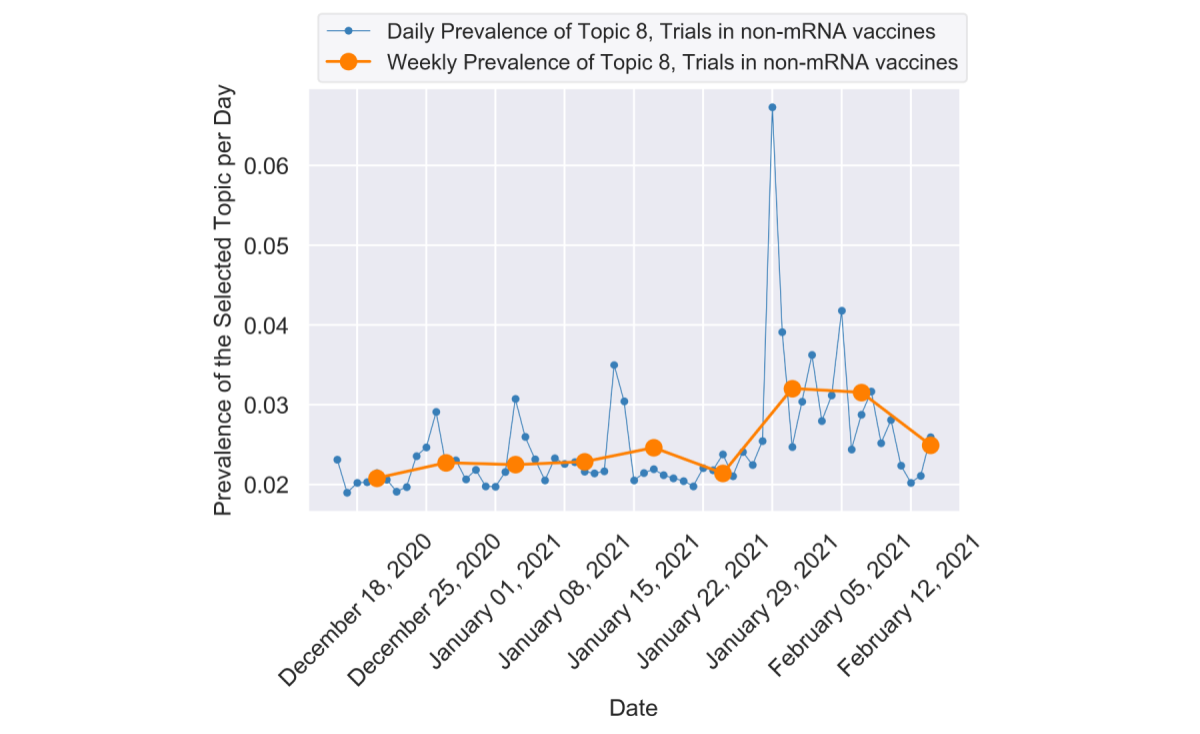
The daily and weekly trends of topic 8.

Figure 4 presents the remaining 6 important topics. Topic 8 (Trials in non-mRNA vaccines) and topic 12 (Low distribution of AstraZeneca vaccine) had apparent spikes on January 29, 2021. For the peak of topic 8 (see Figure 5), we found that the emerging event “the positive trial results of Johnson & Johnson’s single-shot vaccine” caught the public’s eye and stimulated discussion. The relevant contents were tweeted frequently at that moment, and most of them cited news sources [52-54]. The sample tweets were as follows:

…Single-shot Johnson & Johnson vaccine 66 percent effective against moderate and severe illness

cited from Washington post, January 29, 2021

…Johnson & Johnson says its single-shot vaccine is 66% effective overall at preventing moderate to severe illness

cited from Fox8live, January 29, 2021

…Johnson & Johnson’s one-shot #COVID19 vaccine is effective against severe disease

cited from Science News, January 29, 2021

**Figure 6:**
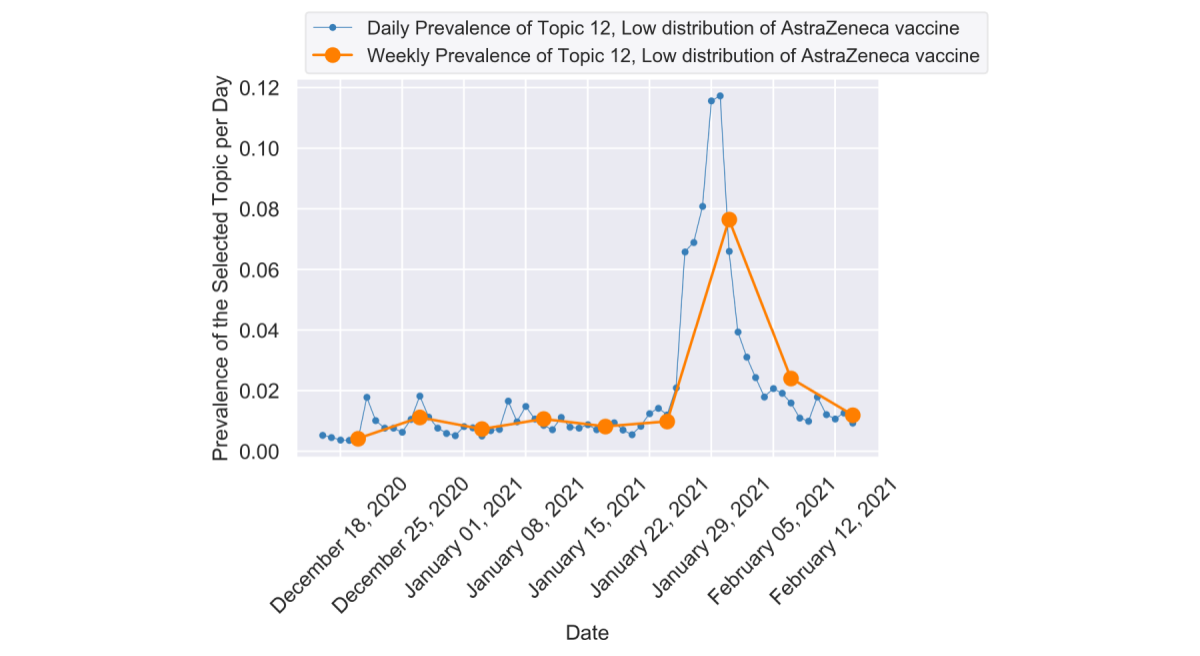
The daily and weekly trends of topic 12.

The spike on Topic 12 (see Figure 6) can be related to the dispute between the European Union and AstraZeneca in the third week of January [55]. The citizens in the European Union expressed their depression about the delay and inefficiency of vaccine ordering, and the representative tweets were as follows:

…EU vaccine delays prompt press frustration

January 28, 2021

…AstraZeneca is supplying European Union vaccine at cost with zero profit. European Union has a cheek to talk about suing AZ

January 29, 2021

…The actions of the European Union to cover their abject failure to obtain vaccine

January 29, 2021

### Tracking Sentiment Over Time

When summarizing the sentiment in all 7,948,886 million tweets throughout the entire period, we observed that the top emotion that appeared in our tweets was fear followed by joy. The percentage of tweets containing each of the emotions from the tweets collected during the entire period is described in Table 2.

**Figure 7:**
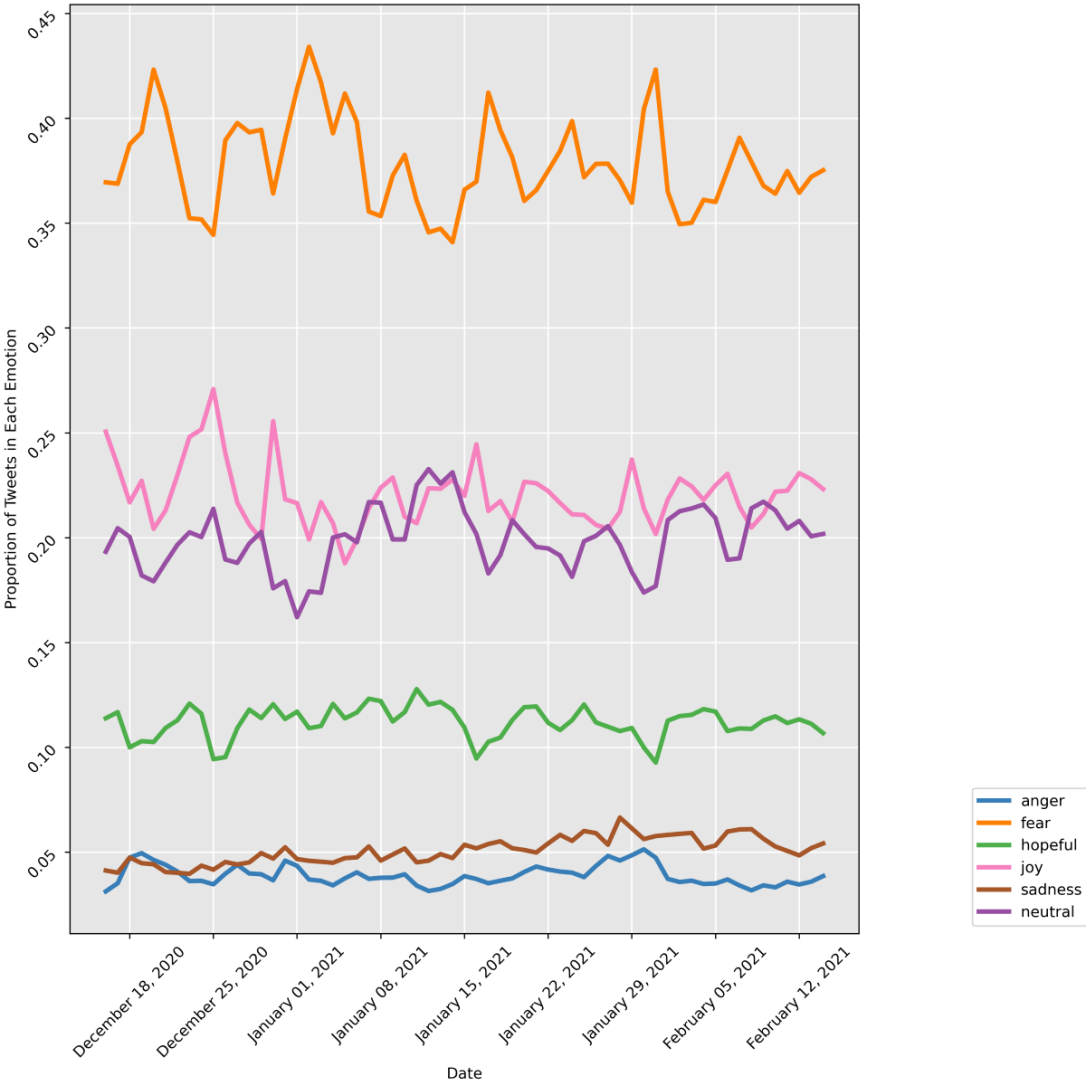
Emotional trends over time.

Figure 7 presents the trends of the 5 emotions during the 60-day period starting from December 16, 2020. It shows that fear was consistently the most frequently detected emotion. Joy was the second most common emotion followed by neutral sentiment. Hopefulness, sadness, and anger were reflected in a lower proportion of tweets. The Augmented Dickey-Fuller test showed that all emotions, except for sadness, were stationary throughout the entire period, while sadness increased throughout the period.

**Table 2:** Proportion of tweets by emotion (N=7,948,886).

Sentiment			Tweet totals, n (%)
**Negative emotion**			
	Fear	3,002,467 (37.8)	
	Sadness	406,095 (5.1)	
	Anger	312,398 (3.9)	
Neutral emotion			1,582,221 (19.9)
**Positive emotion**			
	Joy	1,751,729 (21.9)	
	Hopefulness	406,095 (11.2)	

### Sentiment Trends in the 12 Detected Topics

**Figure 8:**
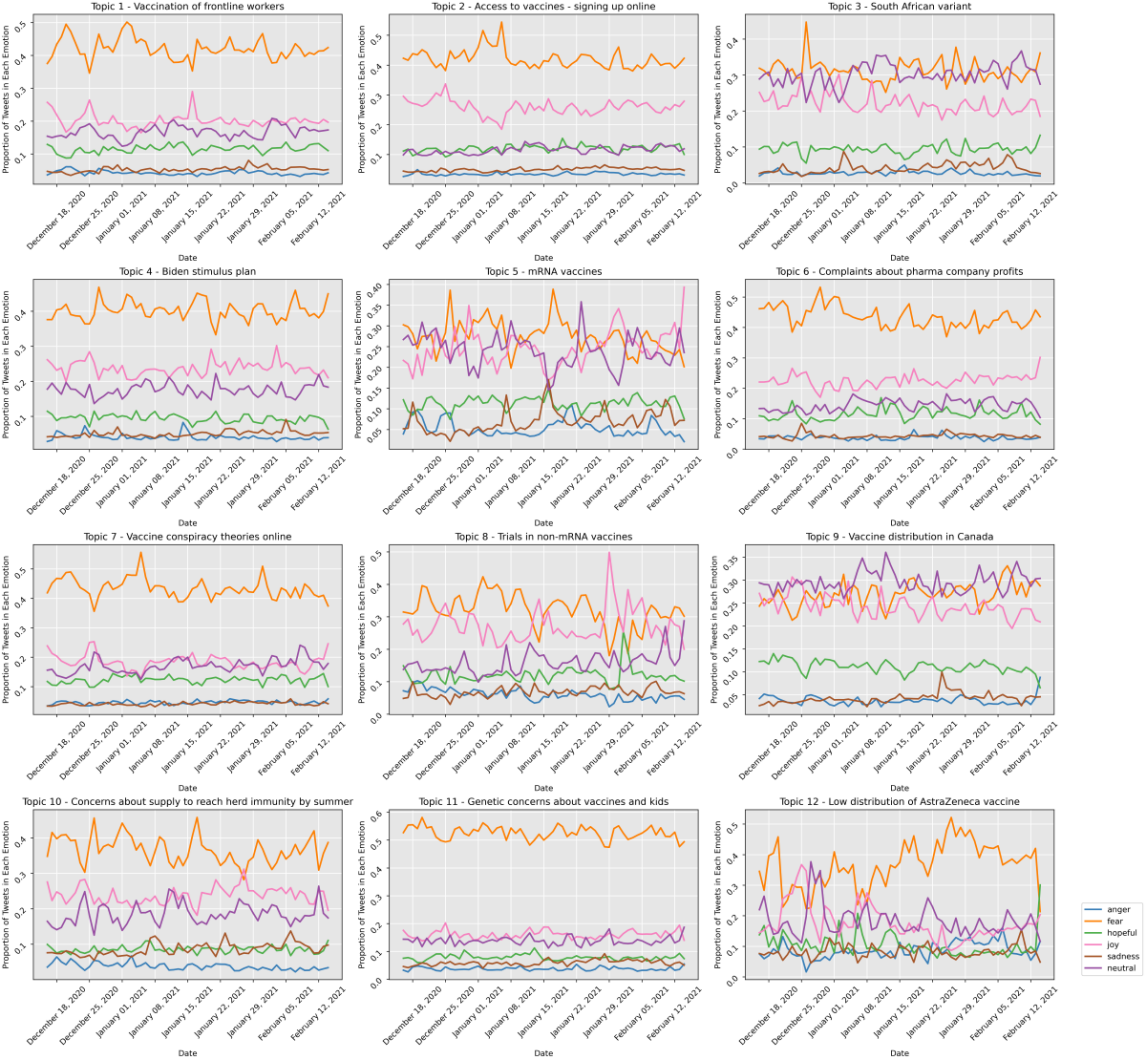
Emotional trends over time regarding topics 1-12.

To analyze the sentiment in each of the top 12 topics, we plotted the proportion of each sentiment for each topic and observed how the percentages changed over time. The percentage of tweets in each sentiment is described in Figure 8.

#### Negative Sentiment

Negative sentiment was the leading sentiment in our tweets, with fear as the leading emotion.

##### Fear

Our graphs show that for the majority of topics, fear was the most observed emotion. In topics 1, 2, 4, 6, 7, 10, and 11, fear was the most observed emotion throughout the majority of the time period. Topic 1 discussed the vaccination of the frontline workers. Some representative tweets from this topic that contained fear were as follows:

…@POTUS Mr. President, I’m really worried about my state (GA) and the rollout with vaccines. There doesn’t seem to be a plan and we are being pushed to have school and teachers are not vaccinated and barely hospital workers and senior citizens have.

February 13, 2021

…@CTVNews Hi, I am an Ontario resident and my wife works at X-ray & Ultrasound clinic in Newmarket. I am worried about her and her Associates not getting the vaccine along with hospital workers, she sees patients every day and I think they must be vaccinated ASAP. Thanks, Charlie

January 12, 2021

The main theme in these tweets was fear that frontline workers would not be vaccinated soon enough and that they would not receive the highest priority in the vaccine rollout.

Topic 2 discussed access to vaccines and signing up online. The most prominent emotion in this topic throughout the period was fear. Below are some example tweets from this topic:

…I got vaccinated. I’m Latino. Making my appt was confusing and my 2nd appt kept getting cancelled even though I work in a hospital. Also lots of fear, distrust and misinformation, people saying the vaccine gives you the 666 sign of the devil, etc. Many people are scared of it. https://t.co/98pguyfuiJ

January 31, 2021

…I’m very concerned my 82-year-old mother must go online to a website; register for the vaccine in Nevada that is still not available until February 28? How do we solve this for our older generation with no computer knowledge to help them get vaccines quicker?

January 06, 2021

We could identify with the struggle to obtain an appointment for vaccinations in many states. There were also technical difficulties with multiple websites that caused concern among many Twitter users.

Topic 4 discussed Biden’s stimulus plan. The plan contained funding for COVID-19 vaccine distribution [56]. In January, the gap between fear and joy widened; however, after Biden took office in January, joy increased and the gap between fear and joy became smaller.

Examples of tweets from topic 4 that conveyed fear are as follows:

…@GovInslee I’m a fan Jay, but I’m worried Washington is going to screw up the vaccine distribution.

January 13, 2021

…@JoeBiden Please save Texas from @GovAbbott’s ignorance and massive logistical failures with respect to distribution of the vaccine

January 17, 2021

Many of the tweets in this topic conveyed fear with respect to not executing Biden’s plan rather than fear of the plan itself.

##### Anger

While fear was the most prominent emotion followed by joy, some topics contained spikes of anger-related tweets. Topic 5 contained a few spikes of anger. Here are some examples of angry tweets from topic 5:

…Coronavirus: European Union anger over reduced Pfizer vaccine deliveries. Why to rely on profiteering Pfizer ? There are other vaccine! https://t.co/E27tWB71IJ

January 15, 2021

…@latimes Is that why it’s killing old people? 20+ dead in Norway alone. Global scientists calling for immediate stoppage of Pfizer drug. Btw it’s not a vaccine by definition. Its mRNA therapy. A vaccine uses a dead virus that’s incubated and cultured.

January 16, 2021

There was anger due to lack of trust of the vaccine manufacturers as well as anger over rumors of deaths and injuries due to the vaccines.

##### Sadness

Sadness was one of the least prominent emotions in our data. It was the highest in topic 10, which discussed concerns about vaccine supply that would enable reaching herd immunity by summer 2021. Here are some representative tweets from this topic containing sadness:

…My dad was so close to getting his vaccine. But he didn’t make it. Meredith pays tribute to her father who died 4 days ago with COVID-19. He was a Cumbrian farmer. She describes him as grumpy but in a charming way. https://t.co/OR5NsNVuZG

January 13, 2021

…@SHCGreen @NicolaSturgeon @jasonleitch @edinburghpaper @lothianlmc @NHS_Lothian @DrGregorSmith Glad to see some people getting the vaccine. Sadly my aunt didn’t get to have hers. Died early hours from COVID. Will miss her very much.

Many of the tweets in this topic containing sad emotion discussed deaths due to COVID-19 that could have been prevented by a quicker vaccine rollout.

Additionally, we saw the following tweets from topic 1 showing sadness:

…Some of these are so painful. 65-year-old local pharmacist, kept working, hence couldn’t social-distance like, well, a writer. Dead as a consequence. Why frontline workers should be further up in the vaccine queue than even 78-year-olds like me. https://t.co/EYZ6uUr5K8

January 21, 2021

…An extended family member was a carer in a home, no vaccine, was in a coma for 2 weeks and passed last week. I didn’t personally know her but her niece is heartbroken. Thought all care home staff had the vaccine according to the Government.

February 03, 2021

These tweets showed sadness and concern that frontline workers would not be vaccinated soon enough and might contract COVID-19.

#### Neutral Sentiment

Many neutral tweets contained information from news websites or from official sources. As a result, we observed that many of these tweets contained links or media. Neutral sentiment was not the leading emotion in any of the topics; however, we still detected many neutral tweets in all topics. Below are the tweets from different topics containing neutral sentiment from the top 6 topics:

Topic 1:

…Westminster residents ages 65 and older are now eligible to receive the COVID-19 vaccine. Read the full press release below for instructions. #westminsterca #covidvaccine #orangecounty https://t.co/7cgiOLQLl5 (January 13 2021)

After 40 hours of work, the volunteers of Broadbent Arena, in Louisville, KY, are eligible for their own vaccines. Every day, the oldest volunteers with 40 hours under their belts get the leftover doses. https://t.co/tB3NY2ECSE (February 04 2021)

Topic 2:

…#Health care workers, anyone 70 years and older, and state/local government employees and contractors who perform #COVID_19 vaccinations and testing in SC can make appointments to get a #vaccine. https://t.co/65iyk1qJWi

January 15, 2021

…The fastest way to register into this system will be online, WV rolling out new vaccine registration system https://t.co/gTzl9s54vq

January 22, 2021

Topic 3:

Virus Updates: S. Africa Halts AstraZeneca Shot; COVID Reinfections May Be Overlooked https://t.co/VRvgEd0DDV (February 08 2021)

Moderna says it’s working on COVID booster shot for variant in South Africa, says current vaccine provides some protection https://t.co/UQLInvRVoO

January 25, 2021

Topic 4:

…COVID-19 vaccine distribution ramps up for 20 million to be immunized by the start of the new year https://t.co/zWUVzjxTNw

December 21,2020

…The $900 billion stimulus package includes unemployment support of up to $300 per week. The bill also includes $45 billion in support for transportation, $82 billion for schools, $20 billion for coronavirus vaccine distribution and $25 billion in emergency assistance to renters.

December 20, 2020

Topic 5:

…Sir Ian McKellen says he feels ‘euphoric’ after receiving the Pfizer/BioNTech vaccine; https://t.co/Jr4XvRUDlH

December 17, 2020

…The @nytimes reported Pfizer announced that they will ship fewer vials of their coronavirus #vaccine to the US, in response to the FDA approving a change to the label saying the vials contain six doses rather than five: https://t.co/w8pmbwWBoB

January 25, 2021

Topic 6

…Column: Pfizer, Moderna expect billions in profits from COVID vaccines. That’s a scandal https://t.co/LIhZT0uTlB

January 04, 2021

…The pharmaceutical company expects around $15 billion of revenue from sales of its COVID-19 vaccine this year, while Wall Street had anticipated $12.7 billion. https://t.co/KkjT4vur1d

February 02, 2021

In all topics, there were a multitude of articles and opinion pieces from different media outlets. The articles typically followed the theme in the topic to which they were classified.

#### Positive Sentiment

Positive sentiment was the second most common in our data and contained 2 emotions: joy and hopefulness.

##### Joy

In topics 3, 5, 8, and 9, the leading emotion fluctuated throughout the time period. While joy was not the leading topic throughout the entire period, in these few topics, the expression of joy exceeded fear for at least some days during the period.

Topic 5 discussed mRNA vaccines. The vaccines discussed in this topic were only the Pfizer and Moderna vaccines since they were given emergency use authorization for use at the time of data collection.

Examples of tweets from topic 5 that contain joy are as follows:

…Congratulations! Still wear your mask and wash those hands, keep yourself safe! I get my second one tomorrow. Moderna or Pfizer? I got the Pfizer, people I know who have gotten their second dose are having a rough couple days. Molly must be so happy!

February 06, 2021

…Pfizer and Moderna seem to be the clear vaccine winners

January 29, 2021

…Wow vaccine is looking awesome. I’m super impressed with Moderna and Pfizer-- and in record time:)

February 13, 2021

Topic 8 discussed trials of non-mRNA vaccines. While there were many days where fear was the top emotion in this topic, joy was a prominent emotion in the tweets discussing this topic since it was the leading emotion in some days during the time period. Below are examples of tweets containing joy from topic 8:

…Waking up to great news on the COVID vaccines front: Novavax 89% efficacy, Johnson&Johnson single dose, and 100% protected from death 28 days after single shot, AstraZeneca fully approved in EU. #VaccinesSaveLives

January 30, 2021

…I participated in the Janssen/Johnson & Johnson #ENSEMBLE2 COVID-19 vaccine trial Only time will tell whether I received vaccine or placebo. But so happy to be taking part. Thanks to all the amazing staff at St. Thomas’ Hospital London @GSTTnhs #janssen # COVID-19 https://t.co/brHCDOJC6u

January 13, 2021

The possibility of having a variety of vaccines that were approved was a cause for joy for many Twitter users.

##### Hopefulness

Topic 12 contained a spike of hopefulness in late December. This topic discussed the concerns of low distribution of the AstraZeneca vaccine. Below are examples of hopefulness in topic 12:

…Hopefully the Oxford vaccine can help out those countries, not just in EU, who don’t have enough vaccines. https://t.co/BrC3dJ71tN

December 21, 2020

…@ChristinaSNP What a smashing day. Sun is shining, a British vaccine for COVID is approved. The European Union approved #brexit deal is being flown in at the moment. When signed the @theSNP can surely let us know their plans for our future, not merely criticize others like #NoDealNicola #BetterTogether

December 30, 2020

We can see that there was some hopefulness regarding the distribution of the AstraZeneca vaccine. However, hopefulness was not the leading emotion during that time period. Additionally, by the end of the time period, fear was by far the most prominent emotion.

## Discussion

### Principal Results

Our study aimed to detect the topics and sentiments of public concerns of COVID-19 vaccines by performing a trend analysis on tweets collected for a period of 60 days when the vaccines were started in the United States and to make practical suggestions to address the concerns of different groups in the public as expressed on social media. Approximately 8 million tweets related to COVID-19 vaccines were collected and 12 important topics were selected for analysis. The 3 most important topics with the highest topic ratio were “Vaccination of Frontline Workers,” “Access of Vaccines–Signing Up Online,” and “South African Variant.” The other topics were mostly related to the concerns about the vaccines as well as their supply and distribution. There were also topics related to the stimulus plan, profits of pharmaceutical companies, and conspiracy theories. Through the trend analysis, it was found that the peaks of the topics were impacted by the events reported in the news and spread through social media. The sentiment analysis showed that 46.9% (3,720,960/7,948,886) of the tweets were negative with emotions of mostly fear, followed by sadness and anger, 33.2% (2,645,705/7,948,886) of tweets were positive with emotions of joy and hopefulness, and 19.9% (1,582,221/7,948,886 tweets) of tweets were neutral. Fear and joy were the most detected emotions. Our analysis examined the 6 different sentiments detected in the tweets and their change over time. We observed that the keywords in each topic did not change much over time; therefore, we were able to track our tweets using the same topics throughout the entire period. In some topics, sentiment was stationary throughout the period, while in others, there were significant trends. For example, in topic 3 “South African variant,” we saw an increase in fear and neutral sentiment over the period and a decrease in joy at the same time. Similarly, we saw an increase in fear and a decrease in joy in topic 12 “Low Distribution of the AstraZeneca Vaccine.” Overall, fear was the top emotion followed by joy. Sadness and hopefulness remained low in most topics throughout the entire period.

### Identifying Specific Concerns in Each Topic by Using Emotional Content

The most notable conclusion from the data is that the main reaction to the COVID-19 vaccines on social media was fear. However, we could identify every one of the emotions in each topic. In each topic, we could find tweets related to the topic containing each of the emotions. By looking at the representative tweets for each topic and each emotion, we were able to learn what specific concerns people may have that may lead to vaccine hesitancy. For example, from topic 1, we found that there was fear surrounding the vaccination of government officials prior to frontline workers. By addressing this publicly and assuring the public that the frontline workers would receive their vaccines as soon as possible, this would help to build public confidence in the vaccine rollout. We could also identify tweets that contained sadness to identify further concerns about the rollout to frontline workers and see Twitter users expressing sadness regarding frontline workers possibly dying due to lack of vaccines. This could be addressed by being more transparent about vaccination timelines or by advocating for more vaccine supply. By being aware of specific concerns as they happen (eg, the vaccination of frontline workers), we will be better able to address the source of concern and reduce vaccine hesitancy.

### Vaccine Administration

The very first dose of the mRNA COVID-19 vaccine by Pfizer and BioNTech was given to a health care worker on December 14, 2020. This may explain why the most significant topic at the start of the study was vaccination of frontline workers (topic 1). As more vaccines were administered, reports of anaphylaxis began to surface, especially with the Moderna vaccine [57]. In the United States alone, 10 cases of anaphylaxis were reported after 4,041,496 (0.002%) vaccines were given between December 21, 2020, and January 10, 2021. This created fear as indicated in the trend, and fear dominated all other emotions throughout the course of the study period. It will be interesting to find out how many of these tweets are from health care personnel versus that from the general public. According to the Centers for Disease Control recommendation, both health care personnel and residents of long-term care facilities were the first to be offered the COVID vaccine [58]. Health care personnel include both clinical and nonclinical staffs such as those who work in food, environmental, and administrative services. It can be assumed that clinical staff have adequate knowledge of vaccines and need not to be afraid to take it. Therefore, public health authorities and health care systems can focus on educating the adverse effects of the vaccine to the nonclinical staff and the general public. For example, anaphylactic reactions occur mostly in people who have a similar reaction to other food and drugs, and it usually occurs within minutes after injection. Better understanding of the adverse effects will minimize fear of the vaccine and thus reduce vaccine hesitancy.

### Access to Vaccines

Signing up online (topic 2), vaccine distribution in Canada (topic 9), and low distribution of AstraZeneca vaccine (topic 12) can all be categorized as accessibility of vaccines. A good amount of positive emotion all through the study period in topic 2 indicated that there was a sense of hope in the midst of the daily rising COVID cases. There is still a large amount of fear regarding COVID-19. It may be the fear of the inability to obtain an appointment for the vaccine. Unlike the United States, Canada does not have her own domestic manufacturers to produce vaccines. As a result, Canada relies on international vaccine manufacturers. The advance purchase contract was signed but there was no specific date for delivery except for “first quarter of 2021.” There was a shortage of supply of vaccines in Canada because of which the Canadian government prioritized giving the first dose to the population first and the second dose 16 weeks later [59] as opposed to after 3 or 4 weeks. The European Union was furious when in early January, AstraZeneca announced that there would be 60% fewer doses of vaccines than it had promised to deliver in the first quarter of 2021. The spikes of fear and anger emotions during this period in topic 12 were the direct reflection of this news. Being able to have access to the vaccines is important once COVID-19 vaccines are authorized for emergency use. Therefore, public health authorities must have plans to work with vaccine manufacturers to manufacture and deliver the vaccines in a timely manner. The transparency of the access information from social media and public health officials is helpful to reduce the fear and anger in the public.

### Practical Implications

In December 2020, the World Health Organization released a safety surveillance manual for COVID-19 vaccines. This manual addressed a number of topics with regards to vaccine administration, including how to communicate information regarding the vaccine on social media [60]. Among other points, the report offers proposals to listen proactively and craft tailored messages to different audiences and address specific concerns of different groups. Using this research, we can take the World Health Organization’s recommendations to provide more specific advice to clinicians and policy makers. To address specific concerns, we divided the 12 topics into 3 groups: favoring vaccines, vaccine hesitant, and vaccine opposed.

#### Favoring Vaccines

The topics that leaned toward those who favor vaccines were topic 1 (vaccination of frontline workers), topic 2 (access to vaccines–signing up online), topic 9 (vaccine distribution in Canada), topic 10 (concerns about supply to reach herd immunity by summer), and topic 12 (low distribution of AstraZeneca vaccine). While these topics also produced negative feelings of fear, anger, and sadness, these negative feelings were regarding concern about not having enough vaccines or not having access to vaccines fast enough. It is crucial to monitor topics that contain tweets from individuals who do want to get vaccinated and keep them informed. Here are some examples of tweets that conveyed fear or concern by individuals who wanted to get vaccinated:

…Anybody know what’s going on with BAT 24-hour appts? Are they fully back up and running again after being shut down for lack of vaccine? My second shot is at 2:45 a.m. next week, and I’m wary of getting up in the middle of the night to go down there to find them closed.

…To be honest, I’d rather risk my life / keep myself in lockdown, for younger key workers to have the vaccine. They are the ones keeping the country going after all.

…Blocking access to a vaccine that could save my life is, oh I don’t know, attempted murder? So is exhaling their COVID breath around me, but the former is active and so much more egregious. Ain’t nobody got time for that mess.

Identifying the topics that vaccine-favoring individuals discussed was crucial to reducing their concern. In accordance with the World Health Organization document, communication on vaccine availability should be active and frequent. An example of using the analysis from this study to inform the public is looking at the visualizations in real time to produce the right messaging on social media. We observed a spike in the volume of topic 1 in the week of December 18. Figure 8 shows that the leading emotion for that week and topic was fear; further, there was a spike in fear during that week for topic 1. Therefore, it was crucial to post messages on social media that week that address the public fear that health care workers would not have adequate access to vaccines. Another key component in keeping the public informed was updating official websites with vaccine information very frequently. During the early days of vaccination, there was a lack of information in many states about the timeline of vaccination for each risk group. Providing more information on the rollout schedule would help ease the concern of individuals in this group. It is crucial to look at the tweets that convey fear and anger in these topics to create the right messaging and address points that concern this group of the public.

#### Vaccine Hesitant

This group of individuals was the most crucial to reach since they can be persuaded to get vaccinated. Topics that discussed vaccine hesitancy were topic 3 (South African variant), topic 5 (mRNA vaccines), topic 8 (Trials in non-mRNA vaccines), and topic 11 (Genetic concerns about vaccines and kids). Below are examples of tweets of the vaccine hesitant from these topics:

…Just keep in mind that some small percentage of those who received the vaccine did not develop immunity, during the clinical trials. And its effectiveness against variant strains is still not fully known.

…The fact that 3 vaccines all appeared to show lowered effectiveness against the variant from South Africa is not encouraging, and the results Novavax announced Thurs were the 1st to occur outside of a lab, testing how well a vaccine worked in people infected with a new variant.

…There were obviously several people in the United Kingdom who had had a severe allergic reaction to this vaccine and had a history of severe allergic reaction, said Offit Several people!!!!! #vaccine

Like the vaccine favorable group, we should also target this group with facts and do so often. However, with this group, we should focus on messages that can be detected in these topics such as those related to side effects of the vaccine, the efficacy of the different types of the vaccine for the original strain of COVID-19 as well as for variants, and why you can still contract COVID-19 even after being vaccinated. We can craft helpful messaging for this group by looking at the topic and emotion data for these topics. For example, we saw an increase in the volume of topic 3 (South African variant) toward the end of January. The most prominent emotion for that topic during that time was fear. Therefore, we can craft messaging on social media regarding the variant that will help with this fear. As the World Health Organization recommends, we should mainly focus on facts and provide up-to-date information to the public through social media regarding the variant.

#### Vaccine Opposed

This group was the least likely to be persuaded by messaging on the vaccine but should not be ignored. This is because they produce messaging on social media that may convince others. Therefore, we should attempt to counter their messaging with up-to-date and correct information. Topics that contained a large number of tweets from individuals that were vaccine opposed were topic 6 (Complaints about pharma company profits), but we can find a small number of tweets from this group in all topics, particularly in tweets that were labeled angry or fearful. Examples of tweets from this group were as follows:

…We have been here before with the Nazis and Thalidomide yet the whole world rushes to take an untested vaccine. People are dying after having the vaccine yet no enquiries into what happened just a rapid cremation and silence. We should all be very worried.

…I bind you up Satan in the name of Jesus, no weapon formed against us shall prosper, and I mean this vaccine is Satan here. “Mark of the beast” read your bibles people.

…He didn’t take the vaccine! He’s a Eugenics partner with Bill Gates they don’t take their own vaccines! How about some proof! He’s just trying to coverup the ill side effects and deaths that are already happening!

Those who were opposed to vaccines were hard to persuade, but we must spread truthful messages to counteract the messages that they spread. Many of the tweets by these individuals did not even discuss concerns that could be addressed but were more about vaccine refusal and the freedom to refuse vaccines. It is important to amplify stories of those who suffered severe consequences by refusing to take the vaccine. This is mostly for the sake of the vaccine hesitant rather than the vaccine opposed. An example of messaging can be obtained by looking at the patterns for topic 6. This topic was stable over time and did not experience any spikes. Therefore, we should stay consistent with our messaging over time and counteract any information on this topic with facts on a consistent basis as recommended by the World Health Organization report.

### Limitations of This Research

#### Limitations of Twitter

Twitter is a large social network with 353 million monthly active users [61]. While this is a significant number of users, there is no guarantee that Twitter users are representative of the global or the US population as a whole. Mislove et al [62] have investigated the ability of Twitter data to represent the US population and have found that areas that are more densely populated tend to be overrepresented in Twitter. Additionally, Gore et al [63] and Padilla et al [64] found geographical bias in their analysis of Twitter data. Both studies found an overrepresentation of urban areas in the demographic data of Twitter users included in their studies. Given this prior research, we must assume that users from urban areas are overrepresented in this data set as well.

#### Keyword Selection

The keywords that were chosen to generate this data set were selected by the authors. The list of keywords described in the data collection section contains keywords that name the colloquial names for the available vaccines at the time of the study. The list also contains terms such as “vaccine” and “coronavaccine” that were included in order to capture a more general discussion regarding COVID-19 vaccines. The list is not meant to be exhaustive and represents the vaccines publicly available at the start of data collection in December 2020.

#### Duplicated Tweets

Bots posting on Twitter are a well-documented phenomenon [65-67]. One of the issues our study faced was the duplication of content due to bot activity on the topic of vaccines. Other research has documented bot activity on COVID-19 and COVID-19 vaccine misinformation as well [65,68,69]. The main issue this may cause in our analysis is that bot activity may overinflate the importance of certain topics. To combat this, we deduped the Twitter data as part of our analysis and reduced the number of tweets from approximately 20 million to approximately 8 million tweets.

### Conclusion

We used topic detection and sentiment analysis as social media trend analysis to better understand the discourse on COVID-19 vaccines tweets. Using this methodology, we could identify the trending topics that reflected the public concerns on COVID-19 vaccines and their responses to the topics indicated by the polarity and emotions on the sentiments. We found that the administration and access to vaccine were some of the major concerns. While most of the information was received from the internet, they were not directly obtained from the health organization. Misinformation may cause negative emotions. In some cases, conspiracy spreading in social media may cause substantial amount of fear. The findings in social media trend analysis are helpful for the health organizations to develop strategies for better communication to the target groups and assist them in coping with their concerns that cause negative emotions or vaccine hesitancy. Disseminating accurate information of COVID-19 vaccines will reduce the negative emotion caused by misinformation or rumors. A report on COVID-19 vaccines by the World Health Organization suggested careful examination of social media to detect specific concerns regarding the vaccines [60]. By understanding what drives different emotions regarding the vaccines, tailored and targeted communication can be developed to provide authoritative health information, which will be helpful to achieve herd immunity and end the pandemic.

## Abbreviations

BERT: bidirectional encoder representations from transformers
NMF: nonnegative matrix factorization
VADER: Valence Aware Dictionary and sEntiment Reasoner
